# Effect of *Lycopersicon esculentum* extract on apoptosis in the rat cerebellum, following prenatal and postnatal exposure to an electromagnetic field

**DOI:** 10.3892/etm.2013.1123

**Published:** 2013-05-17

**Authors:** SIBEL KÖKTÜRK, MELDA YARDIMOGLU, SAADET D. CELIKOZLU, ELIF GELENLI DOLANBAY, ALI CIMBIZ

**Affiliations:** 1 Department of Histology and Embryology, Faculty of Medicine, Ordu University, Ordu 52200;; 2 Department of Histology and Embryology, Faculty of Medicine, Kocaeli University, Kocaeli 41380;; 3 Altıntaş Vocational School of Dumlupinar University, Altıntaş, Kütahya 43800;; 4 Department of Physical Therapy and Rehabilitation, Faculty of Health Sciences, Zirve University, Gaziantep 27260, Turkey

**Keywords:** *Lycopersicon esculentum*, electromagnetic field, apoptosis, mobile phone, cerebellum

## Abstract

The expansion of mobile phone technology has raised concerns regarding the effect of 900-MHz electromagnetic field (EMF) exposure on the central nervous system. At present, the developing human brain is regularly exposed to mobile telephones, pre- and postnatally. Several studies have demonstrated the acute effects of EMF exposure during pre- or postnatal periods; however, the chronic effects of EMF exposure are less understood. Thus, the aim of the present study was to determine the chronic effects of EMF on the pre- and postnatal rat cerebellum. The control group was maintained in the same conditions as the experimental groups, without the exposure to EMF. In the EMF1 group, the rats were exposed to EMF during pre- and postnatal periods (until postnatal day 80). In the EMF2 group, the rats were also exposed to EMF pre- and postnatally; in addition, however, they were provided with a daily oral supplementation of *Lycopersicon esculentum* extract (∼2 g/kg). The number of caspase-3-labeled Purkinje neurons and granule cells present in the rats in the control and experimental groups were then counted. The neurodegenerative changes were studied using cresyl violet staining, and these changes were evaluated. In comparison with the control animals, the EMF1 group demonstrated a significant increase in the number of caspase-3-labeled Purkinje neurons and granule cells present in the cerebellum (P<0.001). However, in comparison with the EMF1 group, the EMF2 group exhibited significantly fewer caspase-3-labeled Purkinje neurons and granule cells in the cerebellum. In the EMF1 group, the Purkinje neurons were revealed to have undergone dark neuron degenerative changes. However, the presence of dark Purkinje neurons was reduced in the EMF2 group, compared with the EMF1 group. The results indicated that apoptosis and neurodegeneration in rats exposed to EMF during pre- and postnatal periods may be reduced with *Lycopersicon esculentum* extract therapy.

## Introduction

In recent years, the use of mobile phones has increased substantially; thus, there is a requirement for the potential effects on the central nervous system to be investigated. Numerous studies have demonstrated that electromagnetic field (EMF) exposure does not significantly increase the apoptosis rate in the brain cells of rodents (1–4). However, there have also been several studies that have revealed evidence to the contrary (5–8).

Apoptosis occurs in response to a wide range of environmental stimuli (9,10). Caspase-3 is a key protein that is involved in the mechanism for apoptosis in several cell types, including neurons (11), and has been implicated in the processes leading to neurodegeneration (12). An imbalance of caspase-3 levels in the apoptotic pathway may promote certain human diseases. Studies of experimental models have suggested that caspase-3 is a reliable indicator of apoptotic rate (13–16). However, data concerning the effects of EMF on apoptosis are limited (1).

Several studies have investigated the acute effects of EMF exposure during the pre- and postnatal periods (17–20). By contrast, less is understood regarding the chronic effects of EMF exposure. The developing human brain is now regularly exposed to mobile telephones pre- and postnatally (17). However, a World Health Organization (WHO) symposium (18) concluded that, due to a paucity of relevant research, it remained unclear whether the developing brain exhibited an increased sensitivity to EMF exposure. A key recommendation of the symposium was to study the impact of EMF exposure on the developing nervous system in immature animals (19). Thus, the aim of the present study was to determine the chronic effects of EMF exposure on pre- and postnatal rats.

*Lycopersicon esculentum* (tomato) and lycopene consumption have been demonstrated to be correlated with reduced incidences of cancer (21–23), cardiovascular disease (24) and type 2 diabetes (25). Lycopene is a carotenoid present in high concentrations in tomatoes (26), and has been demonstrated to provide protection against the cellular damage caused by reactive oxygen species (ROS) (26–28). Lycopene has a high liposolubility and is capable of passing through the blood-brain barrier (29), suggesting that it may have potential as a treatment for brain diseases (30). Qu *et al* revealed that lycopene protected against trimethyltin-induced neurotoxicity by inhibiting the mitochondrial apoptotic pathway. These results indicated that tomato and lycopene may act as neuroprotective agents, due to their anti-apoptotic, antioxidant and free radical scavenging effects (30). In the current study, we investigated the protective effects of *Lycopersicon esculentum* extract on apoptosis and neurodegeneration in the EMF-exposed rat cerebellum, during pre- and postnatal development.

## Materials and methods

### Animals

Albino Wistar male and female rats were used for the study. The rats were housed individually in cages, maintained under standard conditions, and were fed with standard pelletized food and water *ad libitum*. The study comprised three groups of rats, one control and two experimental (EMF1 and EMF2) groups. In the control group, the rats were kept in the same conditions as the experimental groups, but without exposure to EMF. In the EMF1 group, the rats were exposed to EMF during pre- and postnatal periods (until postnatal day 80). In the EMF2 group, the rats received the same EMF exposure as the EMF1 group; however, they were also provided with daily oral supplements of *Lycopersicon esculentum* extract (∼2 g/kg) during the pre- and postnatal periods. All experimental protocols received full approval from the Animal Ethical Committee of Dumlupınar University, Turkey, No. 02.11.2009/9.

### EMF exposure

A commercially available cellular telephone with Global System for Mobile communications (GSM)-900 digital technology was used for EMF exposure. The cell phones were placed on the inside walls of the cages, and the rats were exposed to the effects of the cell phones throughout the pre- and postnatal periods, until they were 80 days old. During the study, the exposure procedure comprised the phones being in standby mode for the entire day, with the exception of 30 min per day when they were in talking mode. The control group was kept in the same conditions as the experimental groups, without exposure to GSM, in a separate room. Therefore, the effects of the EMF on the control group were prevented.

### Immunohistochemical staining

The rats were perfused first with phosphate-buffered saline under ethyl ether anesthesia, and then with 4% buffered paraformaldehyde. The rat brains were dissected and postfixed in the same fixative. Following fixation, coronal blocks of the cerebellar cortex were embedded in paraffin and sectioned at 5 *μ*m. The paraffin sections were studied with caspase-3 immunostaining, using the avidin-biotin peroxidase (ABC) and horseradish peroxidase-streptavidin methods for anti-caspase-3 rabbit polyclonal antibodies (1:50 dilution; Diagnostic Biosystems, Pleasanton, CA, USA). The paraffin-embedded tissue slices were deparaffinized with xylene, and the endogenous peroxidase activity was prevented by incubation in 0.3% hydrogen peroxide in methanol. The tissue slices were hydrated with graded alcohol, treated with 10% normal serum and then incubated with the primer antibody at 4°C overnight. They were then incubated with biotinylated anti-mouse IgG or biotinylated anti-rabbit IgG for 30 min at room temperature. Subsequently, the tissues were incubated with avidin-biotinylated horseradish peroxidase or streptavidin horseradish peroxidase in 10% normal goat serum for 30 min at room temperature. Following this, the slices were visualized using 3-amino-4-ethylcarbazole (AEC) as a chromogen. The negative controls consisted of tissue sections incubated in the absence of the primary antibody. The sections were then mounted for counting.

### Counting of caspase-3-labeled cells

The presence of cells undergoing apoptosis was determined by the immunohistochemical detection of caspase-3. We randomly selected six 200×200 *μ*m^2^ fields from the three coronal sections through the Purkinje and granule cell layers of the cerebellum for each rat, and the caspase-3-labeled cells were counted.

### Cresyl violet staining

The paraffin sections were stained for RNA/DNA with cresyl violet to reveal the dark neurons. The sections were stained with 0.5% cresyl violet (Sigma, St. Louis, MO, USA), dehydrated in graded ethanol and xylene, and then coverslipped with Permount mounting medium (eBioscience, Inc., San Diego, CA, USA).

### Statistical analysis

Statistical analysis was performed using the computer software program SPSS for Windows (SPSS Inc., Chicago, IL, USA). The cell count values of the groups were analyzed by analysis of variance (ANOVA) with post hoc Tukey test calculations for intergroup comparisons. P<0.05 was considered to indicate a statistically significant difference. The data of this study are presented as the mean value ± standard error of the mean (SEM).

## Results

**Figure 1.**
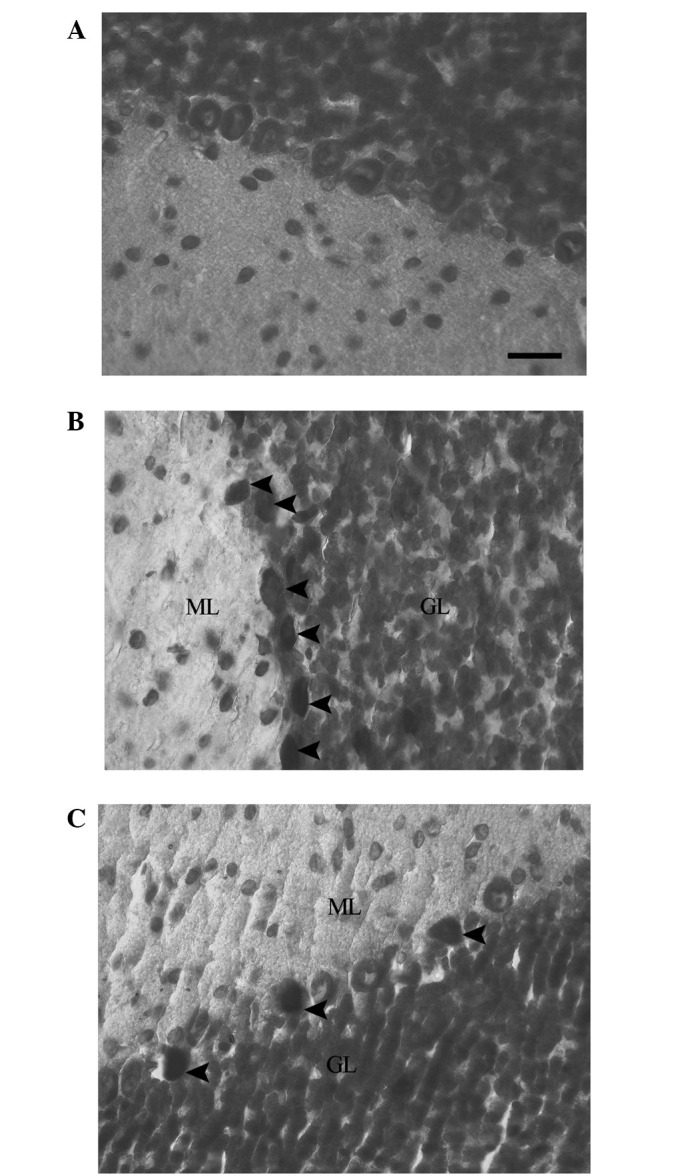
Morphology of the Purkinje neurons. (A) Control group; the normal histological view of the Purkinje neurons. (B) Electromagnetic field (EMF)1 group; the majority of the Purkinje neurons exhibit dark neuron degenerative changes. Features of these cells, such as the shrunken and hyperchromatic (darkly stained) basophilic perikaryon, are indicated in the affected cells by the arrow heads. (C) EMF2 group; a reduced incidence of dark Purkinje neurons, in comparison with the EMF1 group, is evident. A number of dark Purkinje neurons (indicated by the arrow heads) are present, adjacent to the histologically intact neurons. Cresyl violet staining; scale bar, 50 *μ*m. GL, granule cell layer; ML, molecular layer. Cresyl violet staining; scale bar, 50 *μ*m (bar in A calibrates all figures).

Cresyl violet staining was used for evaluation of the presence of dark neurons. The staining of the cerebellums of the rats in the control group revealed normal neuronal morphology in the Purkinje neurons. This was indicated by the pale blue appearance of the Purkinje neurons (Fig. 1A). In the EMF1 group, the Purkinje neurons demonstrated dark neuron degenerative changes. This was indicated by the intensively stained (dark), shrunken and irregular neuronal cytoplasms of the grouped dark Purkinje neurons (Fig. 1B). In the EMF2 group, dark Purkinje neurons were dispersed among intact neurons in the cerebellum. There was a reduced number of dark Purkinje neurons in the EMF2 group, in comparison with the EMF1 group (Fig. 1C).

**Figure 2.**
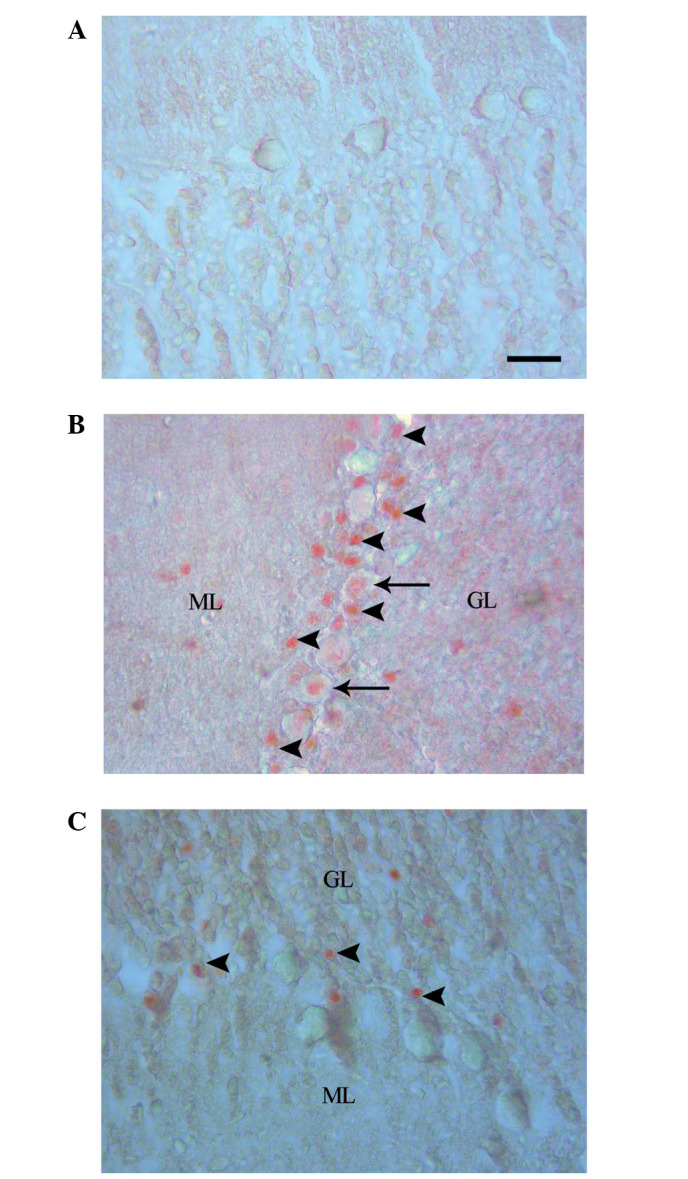
Caspase-3 labeling in Purkinje neurons and granule cells indicates the presence of cells undergoing apoptosis. (A) Control group; apoptotic cells are not present. (B) Electromagnetic field (EMF)1 group; positive staining in the Purkinje neurons (arrows) and granule cells (arrowheads) indicates the marked presence of caspase-3. (C) EMF2 group; positive staining again indicates the presence of caspase-3, although in fewer Purkinje neurons and granule cells (arrowheads) than in the EMF1 group. Caspase-3 staining; scale bar, 50 *μ*m. GL, granule cell layer; ML, molecular layer. Caspase-3 staining; scale bar, 50 *μ*m (bar in A calibrates all figures).

**Figure 3.**
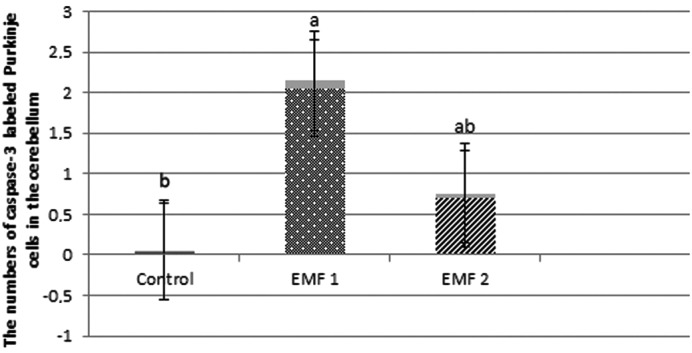
Comparison of the numbers of caspase-3-labeled Purkinje neurons in the cerebellum in the control, electromagnetic field (EMF)1 and EMF2 groups. Values are presented as the mean ± standard error of the mean. ^a^P<0.001 vs. control and ^b^P<0.001 vs. EMF1.

**Figure 4.**
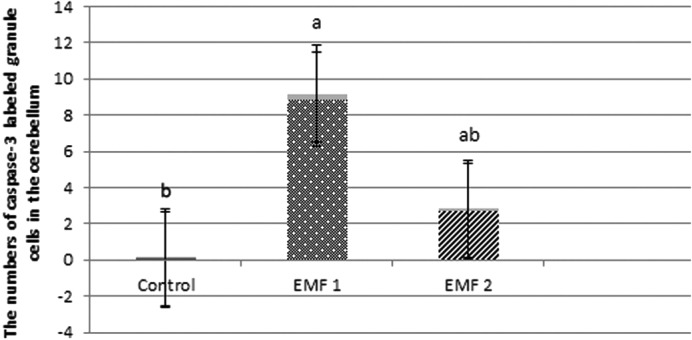
Comparison of the numbers of caspase-3-labeled granule cells in the cerebellum in the control, electromagnetic field (EMF)1 and EMF2 groups. Values are presented as the mean ± standard error of the mean. ^a^P<0.001 vs. control and ^b^P<0.001 vs. EMF1.

Caspase-3 labeling revealed an absence of cell staining in the control group (Fig. 2A), and positive cell staining in the EMF1 (Fig. 2B) and EMF2 (Fig. 2C) groups. The numbers of caspase-3-labeled neurons were observed to be 0.04±0.02, 2.05±0.09 and 0.69±0.06 for the control, EMF1 and EMF2 groups, respectively (mean ± SEM; Fig. 3). In addition, the numbers of caspase-3-labeled granule cells were observed to be 0.07±0.03, 8.86±0.32 and 2.70±0.12, for the control, EMF1 and EMF2 groups, respectively (mean ± SEM; Fig. 4).The caspase-3-labeled Purkinje neurons and granule cells were more prevalent in the EMF1 and EMF2 groups, compared with the control group (P<0.001; Figs. 3 and 4). There was a significant reduction in the number of caspase-3-labeled Purkinje neurons and granule cells in the EMF2 group, as compared with the EMF1 group (P<0.001; Figs. 3 and 4).

## Discussion

EMF exposure has been been demonstrated to induce apoptosis in the human colon (31) and epidermoid cancer cells (32); however, EMF exposure was not observed to induce apoptosis in human peripheral mononuclear blood cells (33) or lymphocytes (34). McNamee *et al* reported that there was no increase in apoptosis in the cerebellums of immature mice subjected to acute EMF exposure (4), whilst Joubert *et al* observed that EMF exposure did not significantly increase the apoptosis rates in rat primary neuronal cultures (3). However, Bas *et al* demonstrated that postnatal exposure to 900-MHz EMF reduced the number of pyramidal cells in the cornu ammonis (CA) of the female rat hippocampus (20). In addition, Sonmez *et al* determined that long-duration exposure to EMF led to a reduction in the number of Purkinje cells in the female rat cerebellum (8). In the present study, it was demonstrated that there was an increase in the number of caspase-3-labeled Purkinje neurons and granule cells in the cerebellums of 80-day-old rats, following pre- and postnatal exposure to 900-MHz EMF. This indicated that chronic EMF exposure had a greater apoptotic effect on the cerebellar cells than short-duration exposure, even at the same frequency.

*Lycopersicon esculentum* and lycopene consumption are correlated with certain diseases that are associated with oxidative stress (21–23). Lycopene, a carotenoid predominantly present in *Lycopersicon esculentum* and *Lycopersicon esculentum* products, has been suggested to exhibit antioxidant activity (35). Several *in vitro* and *in vivo* studies have demonstrated the protective potential of lycopene against oxidative damage (36,37). Qu *et al* observed that lycopene rescued hippocampal neurons from apoptotic cell death induced by trimethyltin, a neurotoxin that partially mimics the pathogenic mechanisms of numerous neurodegenerative disorders (30). Reduced terminal deoxynucleotidyl-transferase-mediated dUTP nick end labeling (TUNEL) staining revealed that pretreatment of the trimethyltin-treated hippocampal neurons with lycopene significantly improved cell viability, by inhibiting neuronal apoptosis. These results indicate that the antioxidant, lycopene, protects against trimethyltin-induced neurotoxicity by inhibiting the ROS-initiated mitochondrial apoptotic pathway. Caspase-3 is considered to be a key protease responsible for a number of the biological and morphological features of apoptosis (38). Türk *et al* demonstrated that lycopene exhibited anti-peroxidative and -apoptotic effects on cyclophosphamide-induced testicular lipid peroxidation and apoptosis (39). The present study revealed that *Lycopersicon esculentum* extract reduced apoptosis in the Purkinje neurons and granule cells in the EMF2 group. This study also demonstrated that there were fewer dark Purkinje neurons in the EMF2 group, in comparison with the EMF1 group. The results indicate that *Lycopersicon esculentum* extract therapy may reduce apoptosis and neurodegeneration in rats exposed to EMF pre- and postnatally.

In conclusion, this study demonstrated that *Lycopersicon esculentum* exerted a protective effect against EMF-induced apoptosis and neurodeneration in rat Purkinje neurons and granule cells, during pre- and postnatal periods. Further investigation is required to evaluate the neuroprotective efficacy of *Lycopersicon esculentum in vivo*.
